# Livestock Density as Risk Factor for Livestock-associated Methicillin-Resistant *Staphylococcus aureus*, the Netherlands

**DOI:** 10.3201/eid1909.121577

**Published:** 2013-09

**Authors:** Peter R. Davies, Bruce H. Alexander, Jeffrey B. Bender, John Deen, Catherine E. Dewey, Julie A. Funk, Claudia A. Munoz-Zanzi, M. Gerard O’Sullivan, Randall S. Singer, Srinand Sreevatsan, Katharina D. Stärk, Mark A. Stevenson

**Affiliations:** University of Minnesota College of Veterinary Medicine, St. Paul, Minnesota, USA (P.R. Davies, J.B. Bender, J. Deen, M.G. O’Sullivan, R.S Singer, S. Sreevatsan);; University of Minnesota School of Public Health, Minneapolis, Minnesota, USA (B.H. Alexander, C. A. Munoz-Zanzi);; Ontario Veterinary College, Guelph, Ontario, Canada (C.E. Dewey);; Michigan State University College of Veterinary Medicine, East Lansing, Michigan, USA (J.A. Funk);; Royal Veterinary College, London, United Kingdom (K.D. Stärk);; Massey University, Palmerston North, New Zealand (M.A. Stevenson)

**Keywords:** swine, livestock, cattle, geographic information systems, methicillin-resistant Staphylococcus aureus, MRSA, cluster analysis, bacteria, zoonoses, the Netherlands

**To the Editor:** We challenge the conclusions of Feingold et al. that “regional density of livestock is a notable risk factor for nasal carriage of LA-MRSA for persons with and without direct contact with livestock” (*1*). They did not study nasal carriage of methicillin-resistant *Staphylococcus aureus* (MRSA), but they retrospectively analyzed 87 culture-confirmed MRSA cases reported to a reference laboratory. These were a mixture of clinical disease isolates and screening (nose, throat, and perineum) isolates that were unevenly distributed between the groups (*2*). Because their analysis aimed to assess exposure risk by residential location, they should have excluded the 5 persons who acquired MRSA outside the Netherlands.

Retrospective case–control studies preclude direct estimation of incidence, prevalence, or risk. However, because of the symmetric property of odds ratios, disease odds ratios can be inferred indirectly from the estimated exposure odds ratios in case–control studies (*3*). However, this case–case study design has no true controls, precluding valid inferences of absolute or relative risks. The higher ratio of livestock-associated (LA)–MRSA to a typeable strain of MRSA (T-MRSA) in rural cases could be attributable to higher risk for LA-MRSA in rural areas, lower risk for T-MRSA in rural areas, or both.

To illustrate this point, suppose urban dwellers had equal prevalence rates of LA-MRSA and T-MRSA of 5%, and rural dwellers had prevalence rates of 2% for LA-MRSA and 1% for T-MRSA. The ratio approach used would indicate that rural dwellers had twice the risk for LA-MRSA than urban dwellers, when the absolute risk is 2.5 times higher in the urban group. At best, their conclusion could be viewed as a hypothesis that should be tested.

Three large community-based studies with better methods collectively refute this hypothesis. Across these studies, LA-MRSA prevalence (44%) was >180 times higher in 352 occupationally exposed persons than in 2,094 rural residents without farm exposure (0.24%) (*4*–*6*). Prevalence in family members of livestock workers was intermediate (5.2%). These consistent observations indicate that exposure to LA-MRSA in livestock-dense regions is a common occupational risk for livestock workers, a lesser indirect risk to their family members, and a negligible risk to persons without livestock or farm contact.

Finally, the contention of Feingold et al. that pig production in the Netherlands is “greatly overshadowed by the density of pig-farming operations in the United States” is mistaken (*1*) (Table). Pig density in the Netherlands is 35 times higher than in the United States, and more than twice that in Iowa.

**Table:** Table. Pig density in the Netherlands, United States (excluding Alaska), and major pig-producing states

Location	No. pigs	Area, km^2^	Pig density, pigs/km^2^	Relative pig density*
The Netherlands	12,100,000†	41,518	291.4	1
United States	67,500,000†	8,108,782‡	8.3	35.0
Iowa	19,700,000	145,744	135.2	2.2
North Carolina	8,600,000	139,393	61.7	4.7
Minnesota	7,600,000	225,174	33.8	8.6

*Pig density in the Netherlands divided by pig density in other locations. †US data were obtained from a quarterly US Department of Agriculture report (http://usda01.library.cornell.edu/usda/nass/HogsPigs//2010s/2012/HogsPigs-09-28-2012.pdf). ‡Alaska was excluded because of minimal swine industry.
